# Integrated characterization and validation of the prognostic significance of microRNA-200s in colorectal cancer

**DOI:** 10.1186/s12935-020-1142-1

**Published:** 2020-02-18

**Authors:** Qiliang Peng, Ming Cheng, Ting Li, Xiangying Chen, Yi Shen, Yaqun Zhu, Bo Xu

**Affiliations:** 1 Department of Radiotherapy & Oncology, The Second Affiliated Hospital of Soochow University, Suzhou, China; 2 Institute of Radiotherapy & Oncology, Soochow University, Suzhou, China; 3 Department of General Surgery, The Second Affiliated Hospital of Soochow University, San Xiang Road No. 1055, Suzhou, Jiangsu 215004 China; 4 Department of Radiation Oncology, The Affiliated Suzhou Science & Technology Town Hospital of Nanjing Medical University, Suzhou, China

**Keywords:** Colorectal cancer, Biomarker, Prognosis, Bioinformatics

## Abstract

**Background:**

Accumulating evidence has demonstrated that microRNA-200s (miR-200a, miR-200b and miR-200c) could serve as promising molecular biomarkers for cancer prognosis. Nevertheless, the associations between miR-200s expression and colorectal cancer (CRC) prognosis remain controversial.

**Methods:**

We applied two mainstream approaches combining meta-analysis and bioinformatics analysis to answer whether miR-200s were associated with the prognosis of CRC patients and why miR-200s could be used as prognostic biomarkers for CRC.

**Results:**

Consequently, low expression of miR-200s was associated with unfavorable overall survival (OS) in CRC patients (HR: 1.09; 95% CI 1.01–1.17; P = 0.025). According to the subgroup analysis, the prognostic role of miR-200s was more significant for tissue samples, large samples, American patients and miR-200a subgroups. Then the target genes of miR-200s were predicted and applied for functional enrichment analyses. The results showed that the target genes of miR-200s were mainly enriched into some vital ontology subjects such as regulation ability, key cell structures and binding function. Moreover, a series of important signaling pathways were identified, which were significantly linked with the initiation and progression of CRC. Additionally, a protein‑protein interaction (PPI) network of miR-200s targets was constructed to screen hub genes and modules. The identified hub genes and modules were validated to be highly involved in the occurrence and development of CRC.

**Conclusions:**

Current evidences revealed that miR-200s could be promising biomarkers for CRC prognosis. However, the findings still need to be validated with more larger-scale prospective studies and biological experiments before miR-200s could be applied into clinical application.

## Background

Colorectal cancer (CRC) remains as one of the most malignant cancer types across almost all the countries and regions because of the marked morbidity and mortality [1]. Despite the advancement of novel treatment options and early detection, the survival outcome of CRC patients has not improved significantly [2]. Current cancer surveillance and management mainly depend on the tumor makers such as CEA and imaging information such as computed tomography (CT) based on tumor node metastasis (TNM) stage and grade [3]. However, more effective prognostic markers and therapeutic methods applied for CRC treatment and prevention have still not been identified, which prevents the precise prediction of the prognosis and metastasis of CRC patients. Thus, there is an urgent need for discovering valuable molecular biomarkers with high precision and noninvasive nature for promoting early screening, prognostic classification, and promising therapeutic strategies for CRC [4].

The discovery of microRNAs (miRNAs), which have been identified as prominent and intriguing tumor biomarkers for noninvasive tests, has provided new ideas for early cancer diagnosis and survival prediction [5]. In general, miRNAs are a class of evolutionarily conserved, endogenous small noncoding with length of 18–24 nucleotides that could influence a variety of critical cellular processes, including cell growth, differentiation, proliferation, apoptosis and metabolism [6]. There is increasing published evidence that deregulated miRNAs with aberrant expression levels were highly involved in cancer initiation and progression and could be a novel kind of biomarkers for various cancer types including CRC [7].

Interestingly, the miR-200s (miR-200a, miR-200b and miR-200c) are typical and most extensively studied examples in functional miRNAs. Recent studies have indicated that miR-200s were dysregulated in various human cancers including breast cancer, ovarian cancer, prostate cancer, esophageal cancer, gastric cancer and CRC [8]. In CRC, there have been several studies validating the association between an aberrant expression level of miR-200s and the survival outcome [9]. Nevertheless, there were still studies revealing the insignificant or opposite results. Moreover, the understanding of the underlying mechanism of miR-200s involved in abnormal development of CRC is still not fully elucidated while this is very important to explain the diagnosis, treatment, prognosis and prevention of this disease.

Therefore, it is essentially necessary to conduct this systematic and comprehensive literature search of all eligible studies for summarizing the published global findings, and obtaining overall prognostic significance of miR-200s expression in CRC. Additionally, we also performed an integrated bioinformatics analysis to uncover the potential mechanisms of miR-200s involved in CRC and to get a better understanding on the biomarker roles of miR-200s.

## Materials and methods

### Literature search strategy

The part of meta-analysis was carried out totally based on the guidelines of Preferred Reporting Items for Systematic Reviews and Meta-Analyses (PRISMA) [10]. To accomplish a comprehensive literature search, we applied several databases including PubMed, EMBASE, Web of science, and the Cochrane Library with the following combination keywords: “microRNA-200a or microRNA-200b or microRNA-200c or miR-200a or miR-200b or miR-200c or miRNA-200a or miRNA-200b or miRNA-200c” and “cancer or tumor or carcinoma or neoplasm” or “colorectal or colon or rectal or rectum”. We conducted the computerized search up to October 26th, 2019. Additionally, the reference lists of the relevant articles were independently scanned for the potentially additional studies.

### Inclusion and exclusion criteria

Studies complied with the following criteria were considered eligible: (1) the study explored prognostic value of miR-200s expression in CRC patients; (2) histologic assessment was used as gold standard for CRC; (3) the study provide prognostic indices such as hazard ratios (HRs) and the corresponding 95% confidence intervals (CIs), or sufficient information to calculate these parameters. Exclusion criteria for the articles included: (1) not related to our research topic; (2) without valuable data for our final data synthesis; (3) the forms of letters, reviews, case reports, conference papers or editorials.

### Data extraction and document assessment

Two researchers independently extracted key information with standardized forms, and discrepancies were settled by a third investigator. The following data and information were collected from each study: first author, publication year, sample source, detection method, patient population, patient number, tumor stage, follow-up time, outcome measures, HR, and corresponding 95% CI. If the HRs and 95% CIs were not directly determined by univariate and multivariate analyses, they may be extracted from provided Kaplan–Meier curves according to methods developed by Tierney et al. [11]. Methodological qualities of selected studies were evaluated by the Newcastle–Ottawa Scale (NOS) method [12]. The NOS scores ranged from 0 to 9; by convention, a study was considered as high quality if it has the NOS score > 6.

### Statistical methods for data synthesis

All of the HRs and corresponding 95% CIs from included studies were applied to estimate the pooled HR for obtaining a whole standpoint of the associations between miR-200s expression and survival outcome. Inter-study heterogeneity was judged by using the Cochran Q and I^2^ test [13]. According to the practice, a P-value of less than 0.05 for the Q test or an I^2^ value of more than 50% was regarded as indicators of high heterogeneity. The random-effects model would be estimated for the studies with a significant heterogeneity (P_Q_ ≤ 0.05; I^2^ ≥ 50%); Otherwise, a fixed effects model would be applied (P_Q_ > 0.05; I^2^ < 50%). Then the subgroup analysis, the meta-regression analysis and the sensitivity analysis were conducted to explore the potential sources of heterogeneity [14]. Finally, Begg’s funnel plot and Egger’s tests were applied to assess the included studies for the possibility of publication bias [15]. All statistical tests in the meta-analysis part were carried out using STATA 12.0 software. A value of P < 0.05 was suggested statistically significant.

### Identification of target genes

The target genes of miR-200a, miR-200b and miR-200c were respectively predicted from the powerful database: miRTarBase, which was designed for comprehensive information regarding experimentally proved miRNA-target interactions [16]. In the most recent update, the database has been recognized as one of the most integrative annotated and experimentally proved miRNA-target interaction tools with more than 13,404 proved miRNA-target interactions from 11,021 articles [17].

### Gene ontology and pathway enrichment analysis

To understand why miR-200s were associated with occurrence and development with CRC, functional enrichment analyses of the targets of miR-200s were performed including Gene Ontology (GO) analysis and Kyoto Encyclopedia of Genes and Genomes (KEGG) pathway analysis [18, 19]. Gene ontology analyses concentrate on three domains including biological processes, cellular components, and molecular functions. To accomplish GO and KEGG analysis of the target genes of miR-200s, the online tool Database for Annotation, Visualization and Integrated Discovery (DAVID) was applied [20]. The DAVID online software identified the significant function terms and potential pathways of the target genes with P‑values < 0.05.

### Construction and analysis of the PPI network

The protein–protein interaction (PPI) information were retrieved from the Search Tool for the Retrieval of Interacting Genes (STRING) database, which is an online tool developed for collecting, scoring and integrating all publicly available sources of PPI data, and complementing these with computational predictions [21]. The target genes of miR-200s were mapped to STRING to evaluate the PPI information and data with confidence scores > 0.4 were regarded as significant and retrieved. Then Cytoscape was employed to analyze and visualize the network. The hub genes were selected using the CytoNCA network analyzer plug-in based on three different centrality measures including betweenness centrality and closeness centrality and degree centrality [22]. Moreover, the MCODE plug-in was utilized to filter and identify the significant network modules in Cytoscape. Finally, KEGG functional analysis was performed to further determine the function of these hub genes and genes involved the identified modules. P < 0.05 was regarded to indicate a statistically significant result.

## Results

### Summary of the included studies

**Fig. 1:**
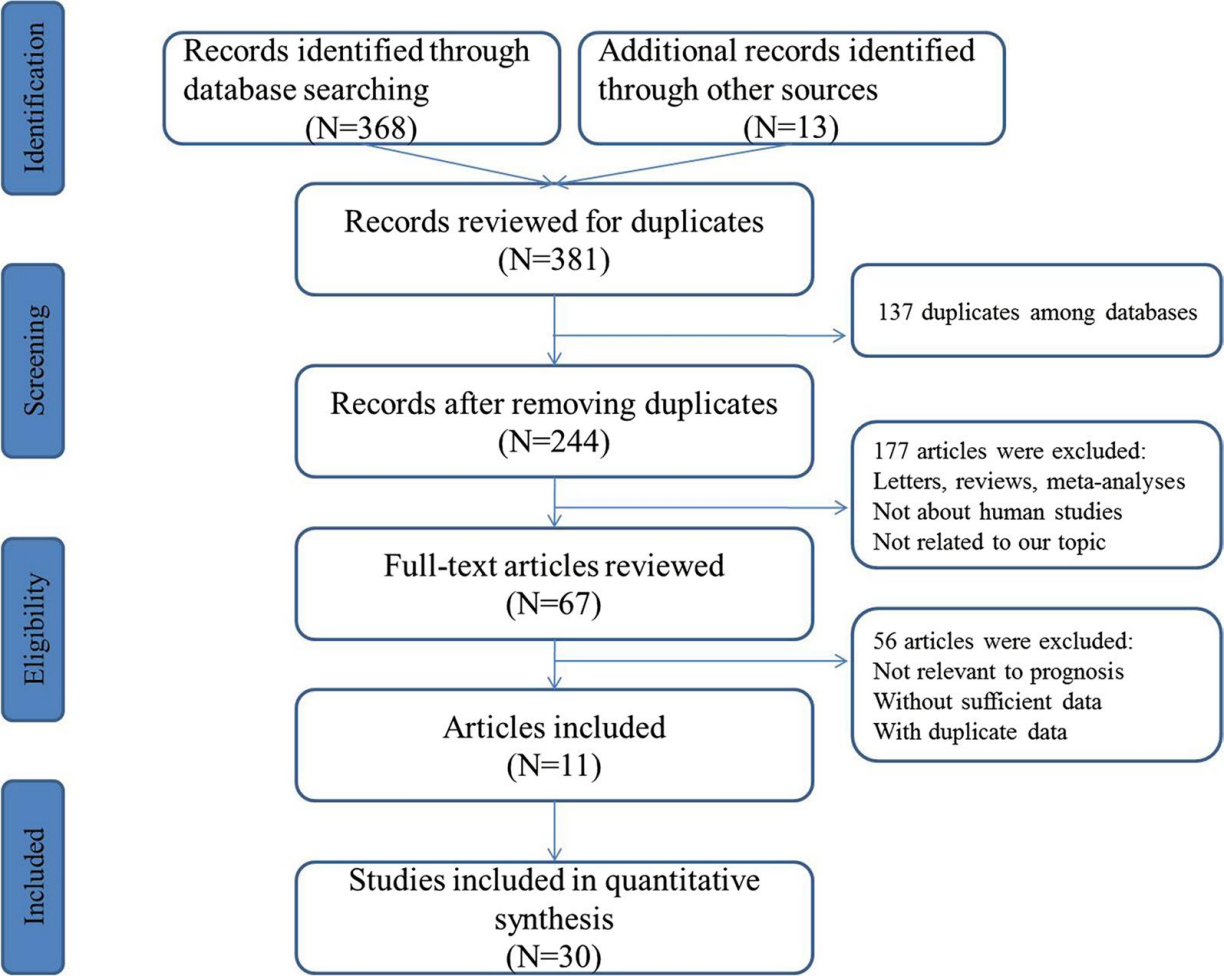
A flowchart presenting the steps of literature retrieval and selection

The literature identification process was depicted in detail at Fig. 1. A total of 381 records were identified through systematic search and manual review for initial search. On the basis of the study selection and exclusion criteria, finally, a total of 11 articles including 30 studies were found to be eligible and enrolled in the data synthesis [23–33]. Overall, all enrolled studies obtained a NOS score more than 6, which revealed a moderately satisfactory quality. The studies were published from 2006 to 2019. The participants of the selected articles were Asians, Europeans and Americans. Most patients had a relatively long follow-up time. The specimens used in these 30 studies were classified as blood (n = 9), and tissue (n = 21). The expression levels of miR-200s were evaluated via quantitative reverse transcription polymerase chain reaction (RT-PCR). The main characteristics of the 16 studies eligible for meta-analysis are shown in Table 1.

**Table 1:** Main characteristics of the studies selected for the meta-analysis

First author	Year	Country	Ethnicity	M/F	N	Age	TNM stage	miRNA	Sample souce	Methods	Endpoints	Median follow-up time	Hazard ratio
Xi	2006	Germany	Europeans	10/14	24	62	I–IV	miR-200c	Tissue	RT–PCR	OS	40	0.68 (0.51–0.92)
Toiyama	2014	Japan	Asians	105/77	182	67	I–IV	miR-200c	Blood	RT–PCR	OS	50	0.37 (0.18–0.78)
Toiyama	2014	Japan	Asians	89/67	156	68	I–IV	miR-200c	Tissue	RT–PCR	OS	50	1.79 (0.91–3.57)
Pichler	2014	USA	Americans	71/40	111	60	II–IV	miR-200a	Tissue	RT–PCR	OS	38	2.04 (1.28–3.25)
Diaz	2014	Spain	Europeans	69/58	127	67	I–III	miR-200a	Tissue	RT-PCR	OS	113	1.20 (1.03–1.40)
Diaz	2014	Spain	Europeans	69/58	127	67	I–III	miR-200c	Tissue	RT-PCR	OS	113	1.12 (1.01–1.25)
Maierthaler	2016	Germany	Europeans	162/146	308	70	I–III	miR-200a	Blood	RT-PCR	OS	72	0.95 (0.71–1.26)
Maierthaler	2016	Germany	Europeans	162/146	308	70	I–III	miR-200b	Blood	RT-PCR	OS	72	1.29 (0.96–1.75)
Maierthaler	2016	Germany	Europeans	162/146	308	70	I–III	miR-200c	Blood	RT-PCR	OS	72	1.19 (0.93–1.52)
Maierthaler	2016	Germany	Europeans	130/89	219	68	IV	miR-200a	Blood	RT-PCR	OS	72	0.81 (0.67–0.99)
Maierthaler	2016	Germany	Europeans	130/89	219	68	IV	miR-200b	Blood	RT-PCR	OS	72	0.83 (0.67–1.03)
Maierthaler	2016	Germany	Europeans	130/89	219	68	IV	miR-200c	Blood	RT-PCR	OS	72	0.87 (0.73–1.03)
Sun	2016	USA	Americans	22/25	47	54	I–IV	miR-200b	Blood	RT-PCR	OS	28	0.38 (0.19–0.77)
Slattery	2016	USA	Americans	401/344	745	64	I–IV	miR-200a	Tissue	RT-PCR	OS	50	1.12 (0.95–1.33)
Slattery	2016	USA	Americans	401/344	745	64	I–IV	miR-200a	Tissue	RT-PCR	OS	50	1.01 (0.93–1.10)
Slattery	2016	USA	Americans	401/344	745	64	I–IV	miR-200b	Tissue	RT-PCR	OS	50	1.11 (0.95–1.30)
Slattery	2016	USA	Americans	401/344	745	64	I–IV	miR-200b	Tissue	RT-PCR	OS	50	1.01 (0.90–1.14)
Slattery	2016	USA	Americans	401/344	745	64	I–IV	miR-200c	Tissue	RT-PCR	OS	50	1.08 (0.90–1.28)
Slattery	2016	USA	Americans	213/183	396	64	I–IV	miR-200a	Tissue	RT-PCR	OS	50	1.18 (0.97–1.41)
Slattery	2016	USA	Americans	213/183	396	64	I–IV	miR-200a	Tissue	RT-PCR	OS	50	1.10 (1.01–1.20)
Slattery	2016	USA	Americans	213/183	396	64	I–IV	miR-200b	Tissue	RT-PCR	OS	50	1.41 (1.05–1.89)
Slattery	2016	USA	Americans	213/183	396	64	I–IV	miR-200b	Tissue	RT-PCR	OS	50	1.09 (0.92–1.28)
Slattery	2016	USA	Americans	213/183	396	64	I–IV	miR-200c	Tissue	RT-PCR	OS	50	1.28 (0.96–1.72)
Roh	2018	South Korea	Asians	67/42	109	68	I–IV	miR-200c	Tissue	RT-PCR	OS	35	0.45 (0.16–1.27)
Shelton	2018	USA	Americans	NA	106	NA	I–IV	miR-200a	Tissue	RT-PCR	OS	120	2.27 (1.27–4.35)
Shelton	2018	USA	Americans	NA	108	NA	I–IV	miR-200b	Tissue	RT-PCR	OS	120	2.17 (1.35–3.57)
Santasusagna	2018	Spain	Europeans	31/19	50	72	I–III	miR-200c	Blood	RT-PCR	OS	45	0.81 (0.67–0.99)
Carter	2019	USA	Americans	112/87	199	66	I–IV	miR-200a	Tissue	RT-PCR	OS	96	2.01(1.09–3.72)
Carter	2019	USA	Americans	112/87	199	66	I–IV	miR-200b	Tissue	RT-PCR	OS	96	1.80 (0.98–3.31)
Carter	2019	USA	Americans	112/87	199	66	I–IV	miR-200c	Tissue	RT-PCR	OS	96	1.97(1.08–3.62)

*F* female, *M* male, *N* number, *OS* overall survival

### Identification of the prognostic value of miR-200s

**Fig. 2:**
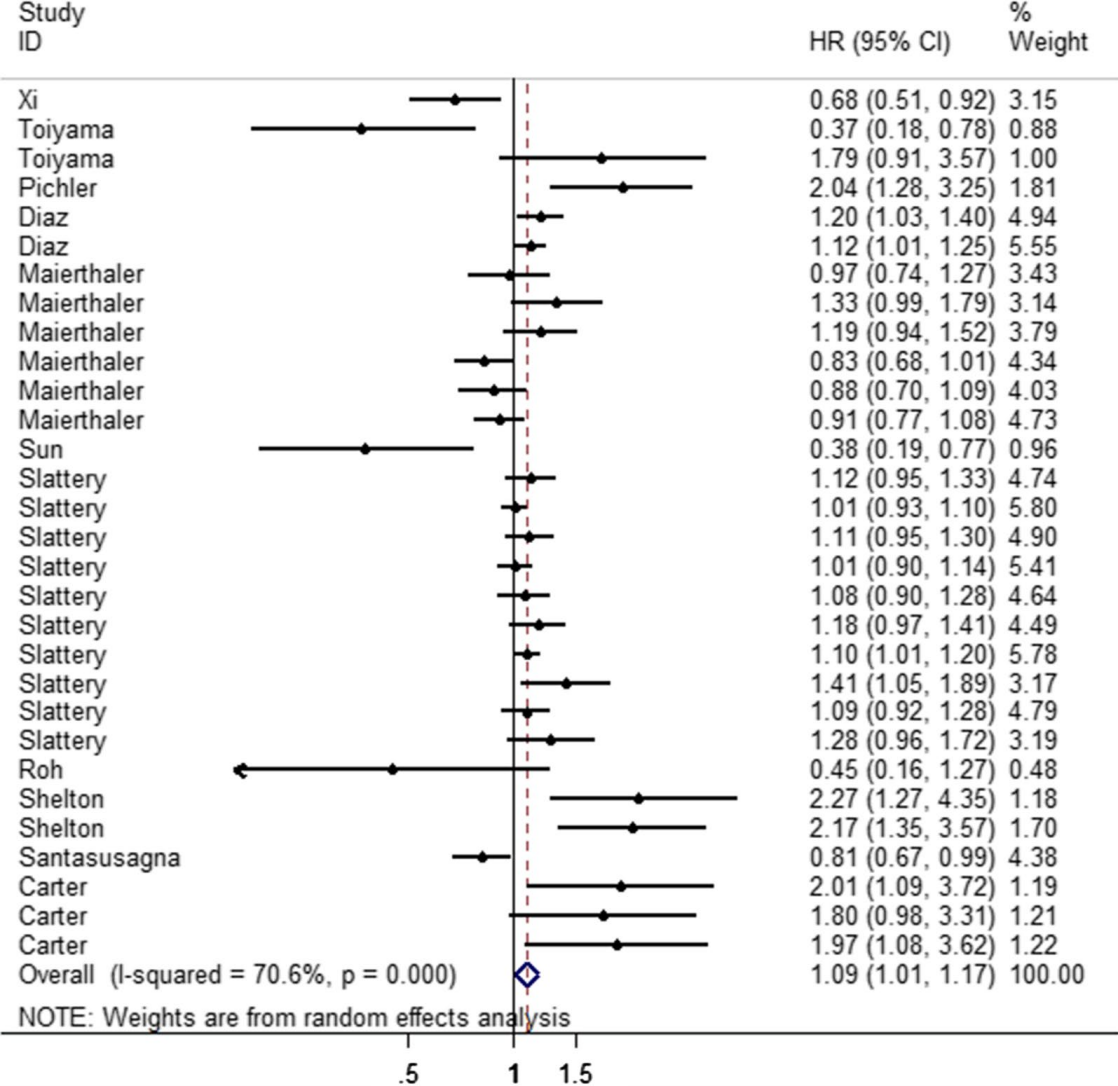
Forest plot of studies evaluating the correlation of low miR-200s expression with overall survival of colorectal cancer patients

As obvious heterogeneity was identified among the enrolled researches (I^2^ = 70.6%, P < 0.001), subsequent meta-analysis with a random-effect model was applied to generate the pooled OS. The results demonstrated that low expression of miR-200s was significantly linked with poor OS (HR: 1.09; 95% CI 1.01–1.17; P = 0.025; Fig. 2), compared than those patients with high expression of miR-200s.

Given that the substantial heterogeneity exhibited, subgroup analysis was performed to explore the heterogeneity of covariates including sample source, sample size, ethnicity and miR-200s classification (Table 2). Sample source subgrouping explored that low miR-200s expression predicted significant poor OS in tissue samples (HR: 1.16; 95% CI 1.08–1.26; P < 0.001), but not in blood samples (HR: 0.89; 95% CI 0.76–1.05; P = 0.161). Subgroups analysis by ethnicity indicated that lower miR-200s expression status was identified as a significant worse prognostic marker in Americans (HR: 1.19; 95% CI 1.08–1.30; P < 0.001), but not in Asians (HR: 0.69; 95% CI 0.23–2.01; P = 0.491) and Europeans (HR: 0.98; 95% CI 0.87–1.10; P = 0.707). Subgroups analyses were also carried out based on sample size and the results revealed that the predictive effect was more significant in large sample size (HR = 1.06; 95% CI 1.01–1.12; P = 0.036) than in small sample size (HR = 1.17; 95% CI 0.94–1.46; P = 0.159). When stratified by miR-200s classification, the prognostic role of miR-200s was more significant for miR-200a with the combined HR being 1.13 (95% CI 1.01–1.26; P = 0.027) than miR-200b (HR = 1.14; 95% CI 0.97–1.33; P = 0.113) and miR-200c (HR = 1.00; 95% CI 0.85–1.17; P = 0.973).

**Table 2:** Results of subgroup and meta-regression analyses

Subgroup	Studies	HR (95% CI)	P-value	Heterogeneity (I^2^) (%)	P_heterogeneity_	Meta-regression (P-value)
Sample source						P = 0.196
Blood	9	0.89 (0.76–1.05)	P = 0.161	68.5	P = 0.001	
Tissue	21	1.16 (1.08–1.26)	P < 0.001	64.4	P < 0.001	
Sample size						P = 0.124
Large (> median)	16	1.06(1.01–1.12)	P = 0.036	40.5	P = 0.047	
Small (< median)	14	1.17 (0.94–1.46)	P = 0.159	82.1	P < 0.001	
Ethnicity						P = 0.136
Asian	3	0.69 (0.23–2.01)	P = 0.491	81.3	P = 0.005	
Europeans	10	0.98 (0.87–1.10)	P = 0.707	72.1	P < 0.001	
Americans	17	1.19 (1.08–1.30)	P < 0.001	67.1	P < 0.001	
miR-200s classification						P = 0.656
miR-200a	10	1.13 (1.01–1.26)	P = 0.027	69.6	P = 0.001	
miR-200b	9	1.14 (0.97–1.33)	P = 0.113	72.3	P < 0.001	
miR-200c	11	1.00 (0.85–1.17)	P = 0.973	74.0	P < 0.001	

Meta-regression analysis was further carried out based on the main characteristics. The meta-regression revealed that heterogeneity may not be induced by sample source (P = 0.196), sample size (P = 0.124), ethnicity (P = 0.136) and miR-200s classification (P = 0.656), as the results have not reached statistical significance.

### Sensitivity analysis and publication bias

**Fig. 3:**
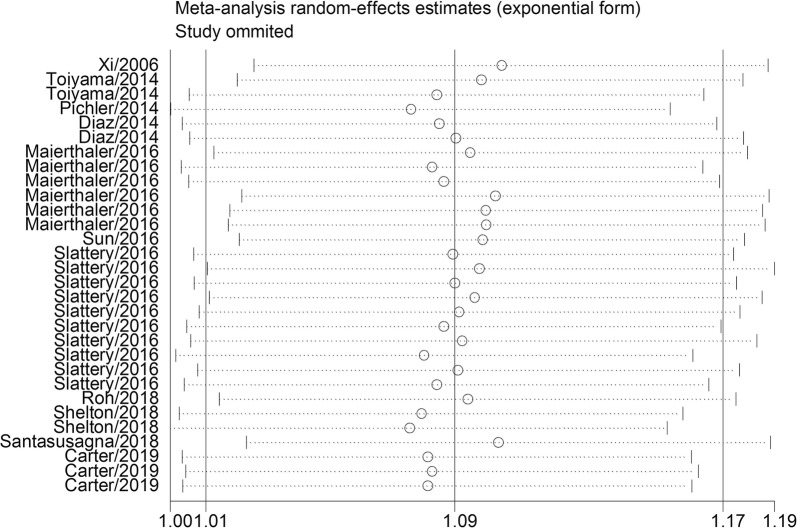
Sensitivity analysis for the influence of individual studies on summarized hazard ratios

**Fig. 4:**
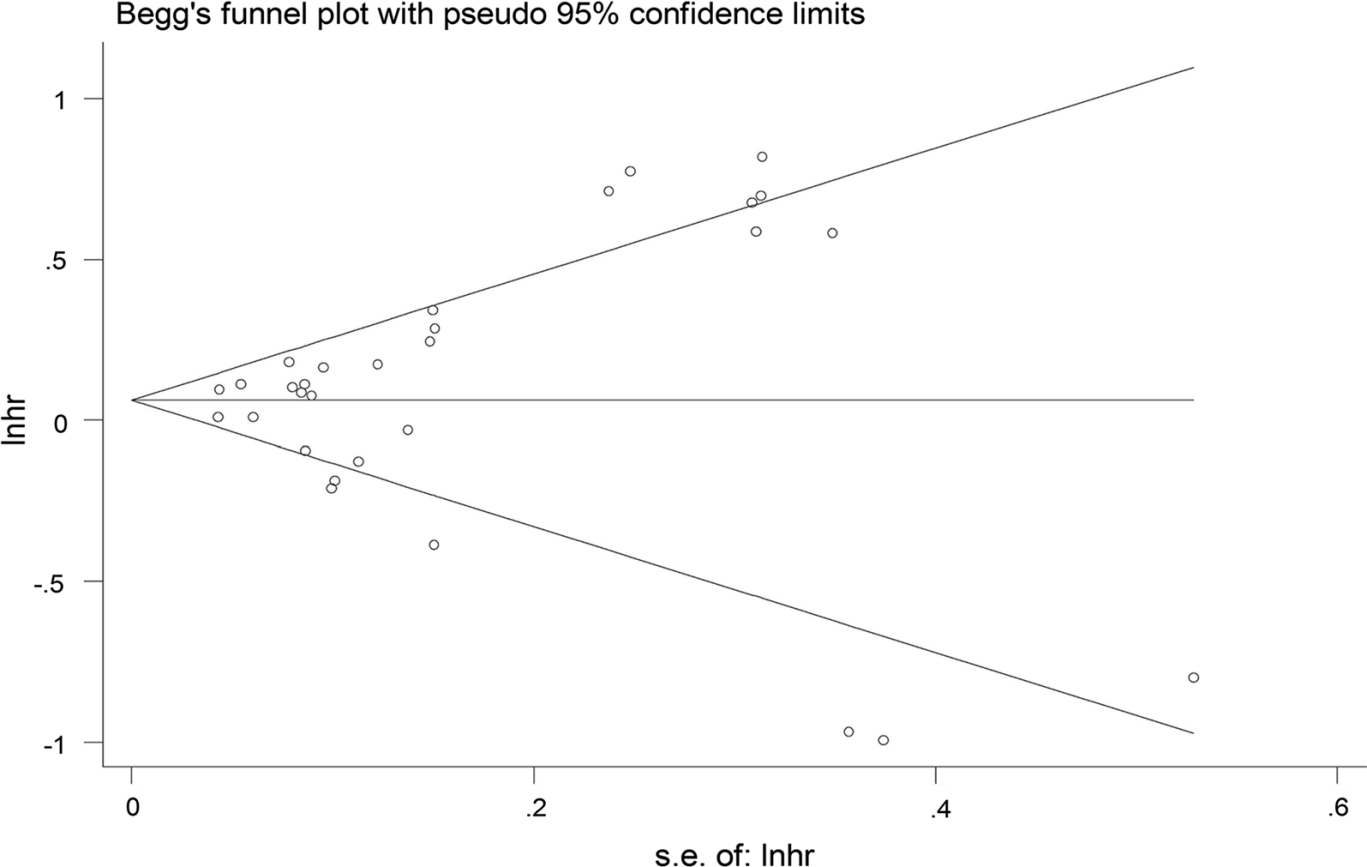
Funnel plot for publication bias analysis

To investigate the robustness of relationship and explore potential factors of statistical heterogeneity, sensitivity analyses were carried out by skipping at a time to evaluate the specific influence of the individual data on the pooled HRs. Sensitivity analysis demonstrated that no obvious variation in pooled HR by excluding any of the study, suggesting that the conclusions are stable (Fig. 3). Begg’s funnel plot and Egger’s tests were employed to evaluate the publication bias. The value was 0.40 for Egger’s tests, which revealed there was no obvious evidence of publication bias (Fig. 4).

### Functional characterization of miR-200s

**Fig. 5:**
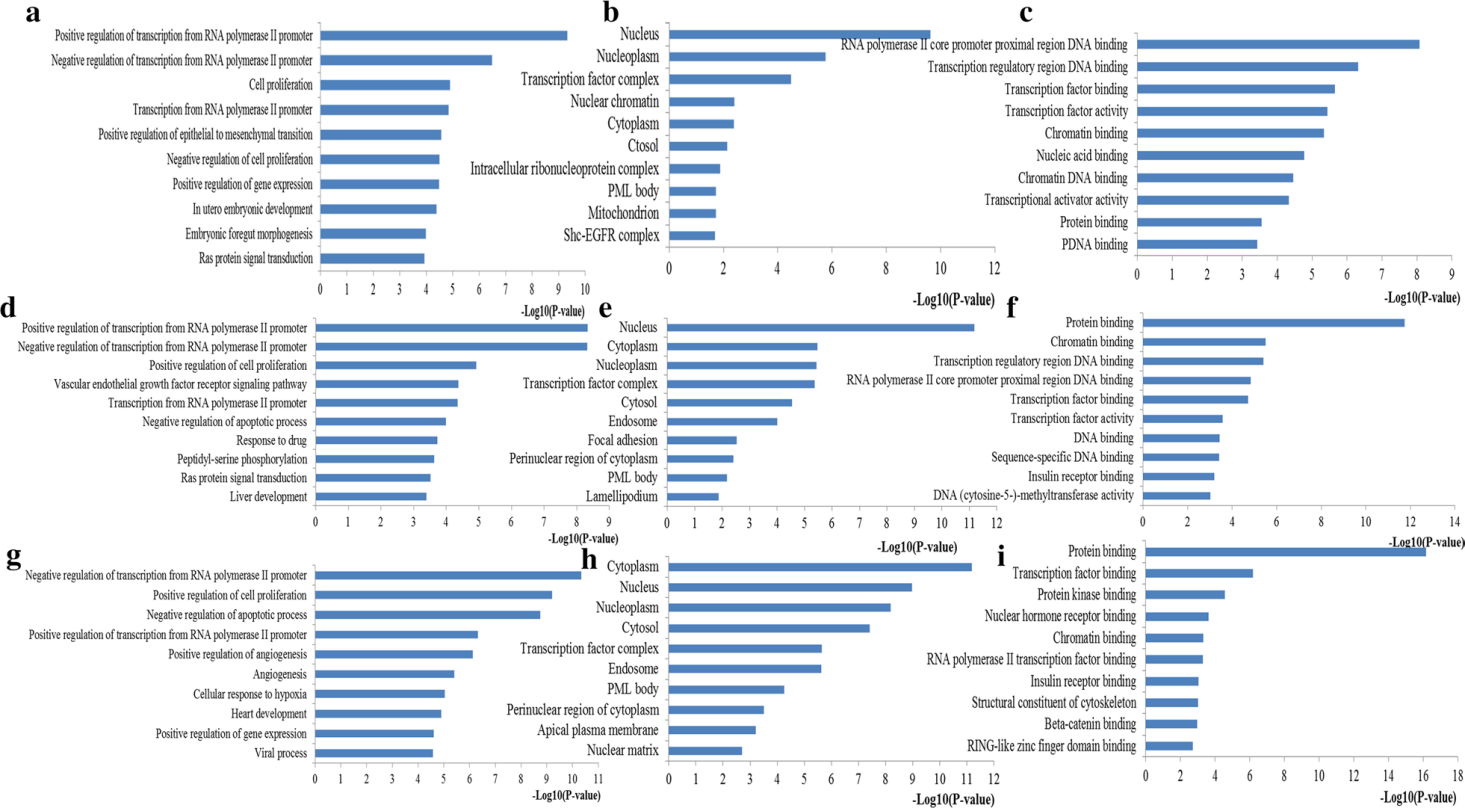
Top ten GO annotation of miR-200s target genes. **a** Biological processes for miR-200a; **b** Cell component for miR-200a; **c** Molecular function for miR-200a; **d** Biological processes for miR-200b; **e** Cell component for miR-200b; **f** Molecular function for miR-200b; **g** Biological processes for miR-200c; **h** Cell component for miR-200c; **i** Molecular function for miR-200c

To understand the miR-200s underlying biological processes and pathways, GO and KEGG pathway analyses were performed to explore the biological functions. The top 10 enriched results of GO analysis were presented at Fig. 5. With respect to biological processes, the target genes of miR-200a, 200b and miR-200c were all primarily enriched in regulating some important biological processes such as regulation of transcription from RNA polymerase II promoter, regulation of cell proliferation and regulation of apoptotic process. Regarding the enriched cellular components, the target genes of miR-200a, 200b and miR-200c were all mainly clustered in some key cell structures including nucleus, cytoplasm and nucleoplasm. In addition, with regard to molecular function, the target genes of miR-200a, 200b and miR-200c were all highly involved in binding function containing transcription regulatory region DNA binding, protein binding, and transcription factor binding.

**Fig. 6:**
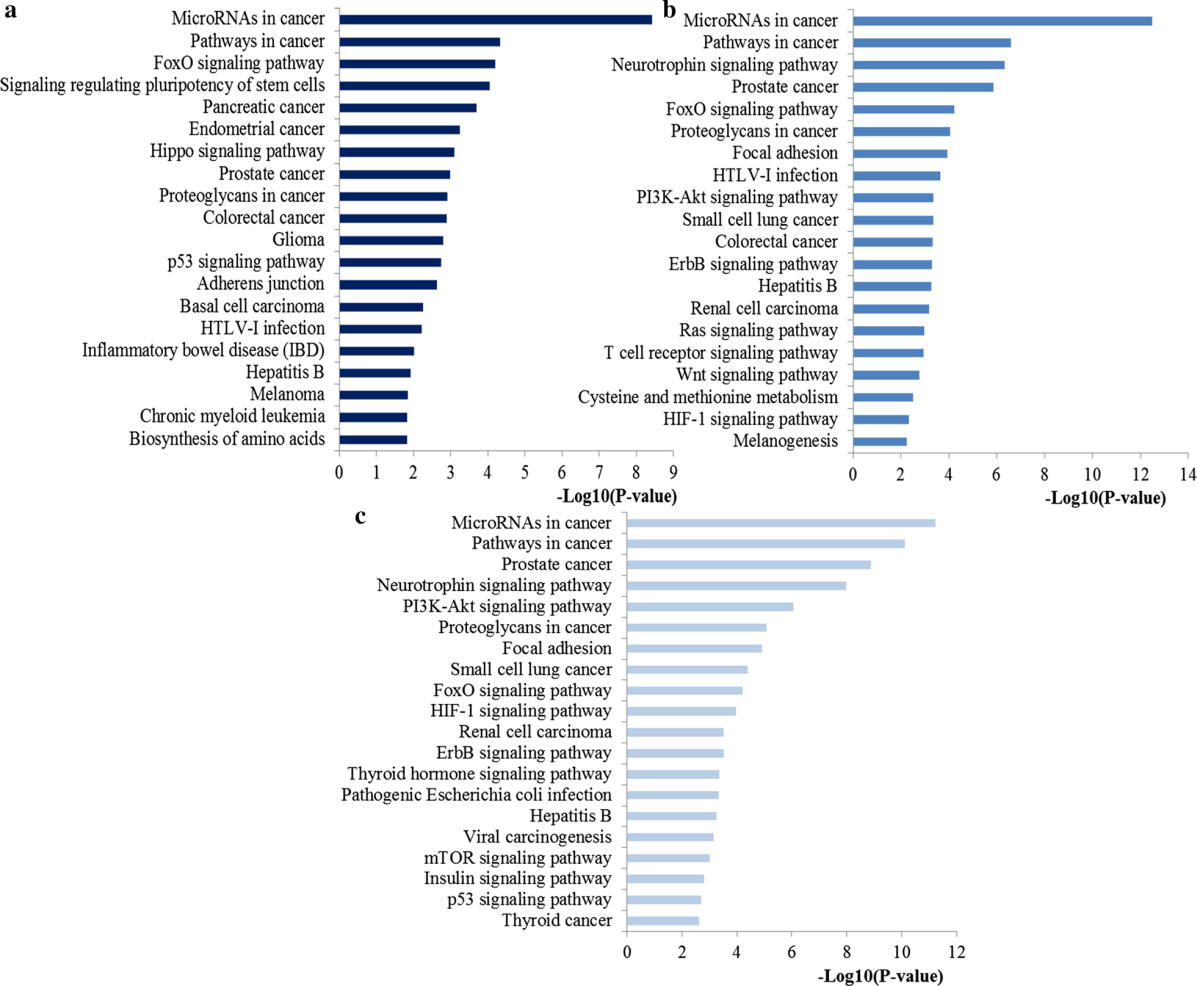
Pathway enrichment results. **a** Top 20 pathways enriched by all the target genes of miR-200a; **b** Top 20 pathways enriched by the hub nodes of miR-200b; **c** Top 20 pathways enriched by the hub nodes of miR-200c

**Fig. 7:**
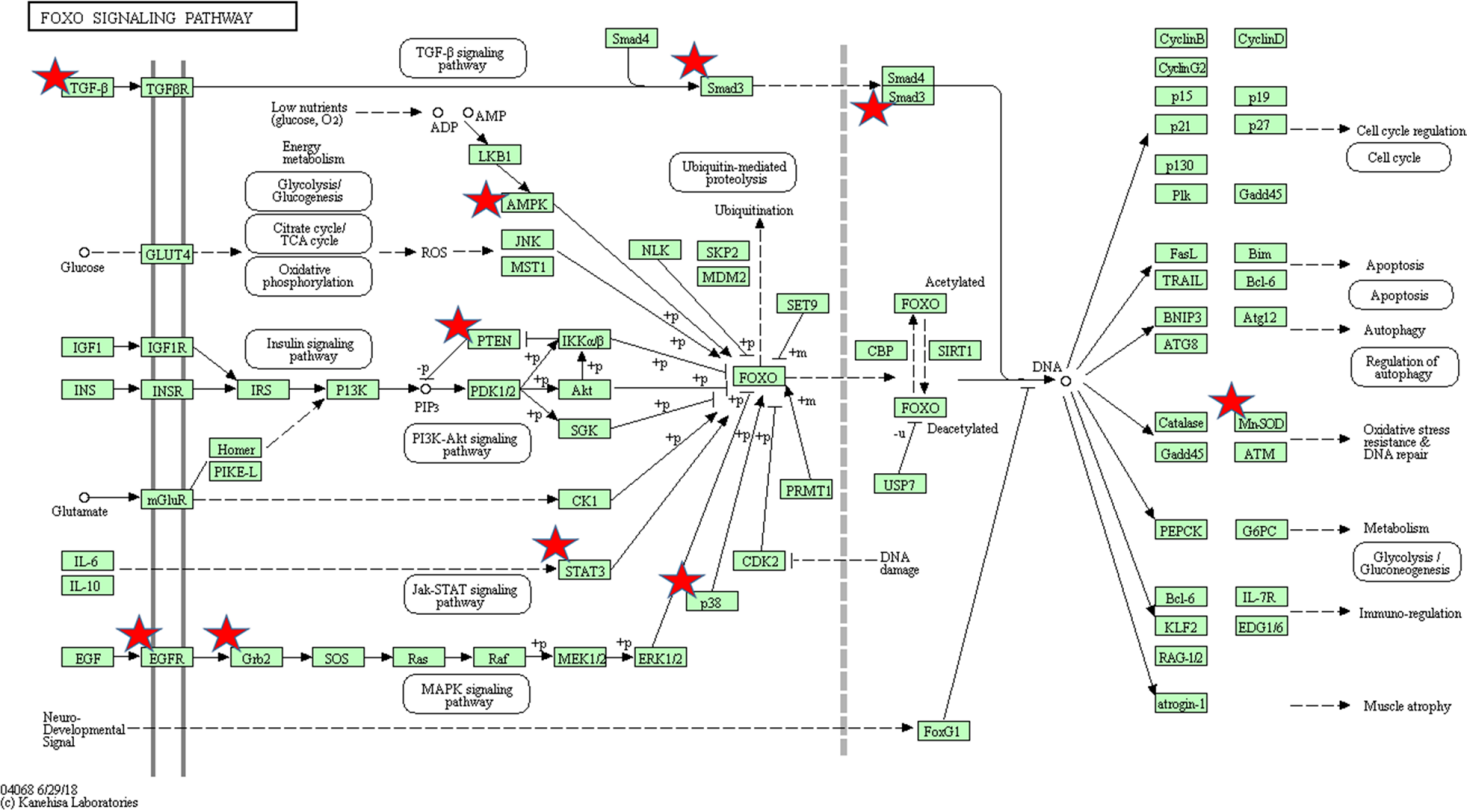
The FOXO signaling pathway enriched in KEGG. Objects with pentagrams are acting locus by mapped genes

The top significantly enriched KEGG pathways for the target genes of miR-200a, miR-200b and miR-200c were also identified and plotted at Fig. 6. These genes may play a crucial role in several important pathways including miRNAs in cancer, pathways in cancer, FoxO signaling pathway, proteoglycans in cancer, colorectal cancer, p53 signaling pathway, PI3K-Akt signaling pathway, HIF-1 signaling pathway and focal adhesion. As shown in Fig. 7, FoxO signaling pathway, one of the most representative pathways, also had close relations with cell cycle, apoptosis, regulation of autophagy, MAPK signaling, PI3K-Akt signaling and JAK-STAT signaling pathway.

### Integration of protein–protein interaction network

**Fig. 8:**
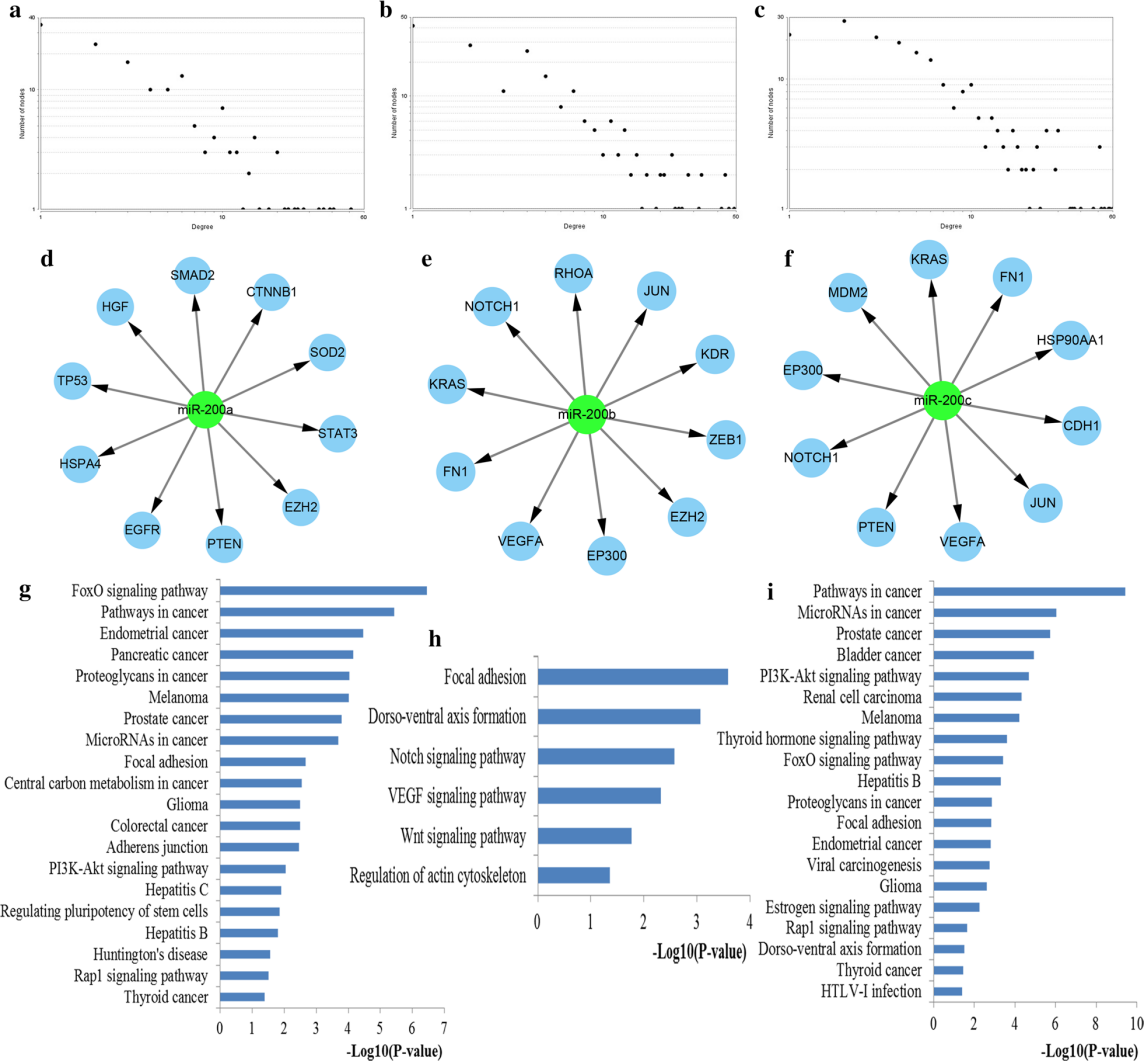
PPI network construction results. **a** Degree distributions of nodes for the network set up with miR-200a targets; **b** Degree distributions of nodes for the network set up with miR-200b targets; **c** Degree distributions of nodes for the network set up with miR-200c targets; **d** Hub genes of the network for miR-200a targets; **e** Hub genes of the network for miR-200b targets; **f** Hub genes of the network for miR-200c targets; **g** Pathway enrichment results for the selected hub genes of miR-200a targets network; **h** Pathway enrichment results for the selected hub genes of miR-200b targets network; **i** Pathway enrichment results for the selected hub genes of miR-200c targets network

To further investigate the potential associations among the target genes of miR-200s, we constructed the PPI networks of the targets of miR-200a, miR-200b and miR-200c, respectively. By separately uploading the targets of miR-200a, miR-200b and miR-200c into STRING database, the PPI networks of miR-200a, miR-200b and miR-200c were visualized using Cytoscape. To identify the most important target genes of miR-200s, the Cytoscape CytoNCA Network Analyzer plug‑in identified top ten hub genes from the PPI networks of miR-200s by estimating the network nodes based on centrality analysis (Fig. 8). In detail, *TP53*, *EGFR*, *PTEN*, *CTNNB1*, *STAT3*, *HSPA4*, *SMAD2*, *EZH2*, *HGF*, *SOD2* were identified for miR-200a, *KRAS*, *VEGFA*, *EP300*, *JUN*, *ZEB1*, *FN1*, *RHOA*, *EZH2*, *NOTCH1*, *KDR* were screened for miR-200b, and *EP300*, *PTEN*, *VEGFA*, *FN1*, *CDH1*, *NOTCH1*, *KRAS*, *JUN*, *MDM2*, *HSP90AA1* were retrieved for miR-200c. Comprehensive function analysis indicated that the hub genes for miR-200a were significantly associated with FoxO signaling pathway, pathways in cancer, proteoglycans in cancer, miRNAs in cancer, colorectal cancer, and PI3K-Akt signaling pathway, the hub genes for miR-200b were mainly involved in Focal adhesion, Notch signaling pathway, VEGF signaling pathway, and Wnt signaling pathway while the hub genes for miR-200c were highly linked with pathways in cancer, miRNAs in cancer, PI3K-Akt signaling pathway, FoxO signaling pathway, Proteoglycans in cancer, Focal adhesion and Viral carcinogenesis.

### Module screening and analysis

**Fig. 9:**
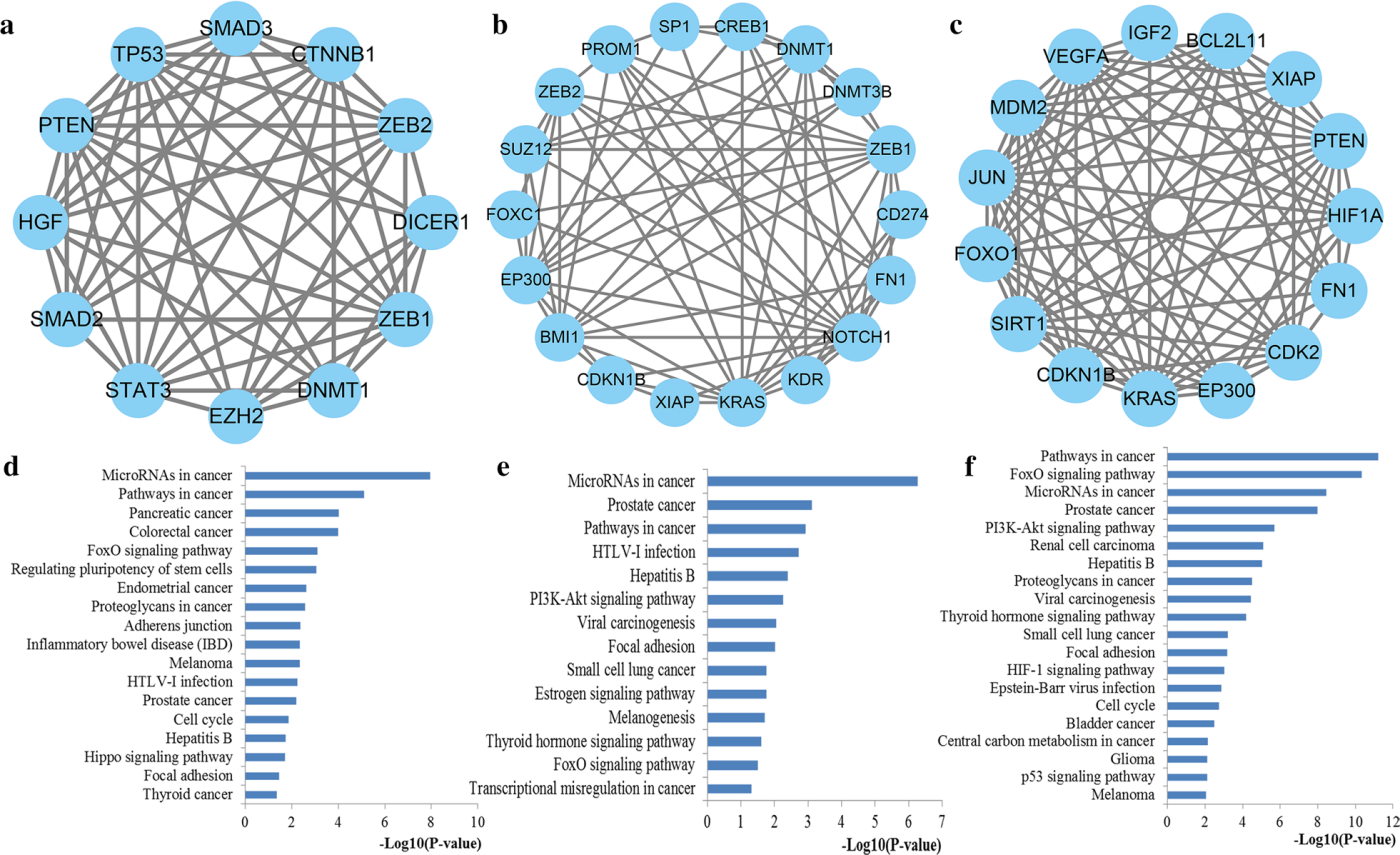
Module analysis results of the PPI network. **a** The most significant module in the PPI network for miR-200a targets; **b** The most significant module in the PPI network for miR-200b targets; **c** The most significant module in the PPI network for miR-200c targets; **d** Pathways enriched by all the nodes involved in the identified module for miR-200a; **e** Pathways enriched by all the nodes involved in the identified module for miR-200b; **f** Pathways enriched by all the nodes involved in the identified module for miR-200c

In addition, the MCODE plug‑in identified the most significant modules, and then conducted function and pathway enrichment analysis (Fig. 9). The most significant module for miR-200a network consisted of 12 nodes and 56 edges, which were primarily involved in miRNAs in cancer, pathways in cancer, colorectal cancer, FoxO signaling pathway, proteoglycans in cancer, cell cycle and Hippo signaling pathway. The most important module for miR-200b network contained 18 nodes and 73 edges, which were mainly linked with miRNAs in cancer, pathways in cancer, PI3K-Akt signaling pathway, focal adhesion, FoxO signaling pathway, and transcriptional misregulation in cancer. The most vital module for miR-200c network including 15 nodes and 86 edges, which were highly associated with pathways in cancer, FoxO signaling pathway, miRNAs in cancer, PI3K-Akt signaling pathway, proteoglycans in cancer, Viral carcinogenesis, HIF-1 signaling pathway, cell cycle, and p53 signaling pathway.

## Discussion

Numerous profiling studies have demonstrated that aberrant expression of miRNAs plays a significant role in the diagnosis and prognosis of human carcinomas including CRC (Additional file 1: Table S1). As one of the most representative miRNA members, miR-200s have been demonstrated by mounting evidence, indicating that miR-200s are noteworthy biomarkers for CRC prognosis. However, inconsistent results have emerged in different studies. Therefore, to clarify the prognostic value of miR-200s as survival prediction biomarkers for CRC, data from published studies of miR-200s were systematically collected and assessed. We separately analyzed the biomarker roles of miR-200s through an integrated bioinformatics analysis to uncover their underlying mechanism involved in CRC.

We first quantitatively explored the prognostic role of miR-200s through a comprehensive meta-analysis. The result indicated that patients with a low miR-200s expression level were significantly correlated with shorter OS, compared than those with a high miR-200s expression level. Thus, it was preliminarily concluded that low miR-200s expression was closely related with worse OS in CRC. Afterwards, the subgroup analysis was selected to investigate the main factors that may contribute to the heterogeneity. The subgroup analysis results suggested that low expression of miR-200s was more significantly associated with worse OS of CRC patients among Americans than Asians and Europeans, which suggested that ethnicity may play an important part in association of miR-200s expression and CRC patient prognosis because of the variations in genetic background, environmental factors. According to the analysis results, it was also summarized that sample size had a significant impact on the predictive role of miR-200s which means that well-designed studies with larger sample sizes are required to further improve the predictive role of miR-200s. Our results also suggested that the prognostic role of miR-200s varied for different types of sample types. Based on the analysis results, tissue samples could be considered as better sample source for classifying miR-200s as the molecular predictive biomarker of CRC. The results from subgroup studies by miR-200s classification revealed that the subgroup of miR-200a emerged statistically significantly higher predictive accuracy than miR-200b and miR-200c. Moreover, the meta-regression was also performed to seek the potential sources of heterogeneity; however, no significant findings were identified. In addition, sensitivity analysis demonstrated the robustness of our study. Promisingly, no obvious publication bias was found.

To further qualitatively explore the potential biological and molecular mechanisms associated with miR-200s, bioinformatics analysis was applied to investigate the underlying functions, pathways, and networks of the genes. The GO analysis indicated that the target genes of miR-200a, miR-200b and miR-200c were mainly involved in regulating some important cellular processes at the biological process level, significantly enriched in some key cell structures for the cellular component level, and highly involved in binding function regarding the molecular function level, which primarily revealed the function of miR-200s involved in CRC initiation and progression. A KEGG analysis further explored the function of miR-200s and revealed that the target genes of miR-200a, 200b and miR-200c were particularly enriched in some important pathways. Most of identified pathways have been demonstrated to play important roles in the occurrence and development according to previous studies. For example, miRNAs in cancer, pathways in cancer, and colorectal cancer pathways intuitively reflected the relationships among miR-200s and CRC. Moreover, proteoglycans have been widely recognized as communication molecules which produce a marked effect in the invasion and growth and metastatic properties of cancerous cells [34]. Accumulating evidence indicates that proteoglycans have a significant impact on the behavior of cancer cells and the microenvironment during the progression of carcinogenesis and may serve as attractive pharmacological targets in cancer immunotherapy [35]. FoxO signaling plays a pivotal functional role as regulator of cellular homeostasis and putative tumor suppressor [36]. Targeting FoxO signaling may represent a potential approach to treat cancer [37]. Focal adhesion kinase, as a tyrosine kinase with high expressions in cancer cells, has been identified highly involved in the progression of tumors to a malignant phenotype [38]. Converging evidence have indicated that p53 signaling play an important role in various transcriptional and non-transcriptional biological processes such as cell proliferation, senescence, DNA repair, and cell death [39]. Previous studies have demonstrated that PI3K-Akt signaling serves as crucial coordinator of intracellular signaling including cell growth, differentiation, migration, and survival, as well as angiogenesis and metabolism [40]. Increasing evidence has confirmed the important part of Wnt signaling in a great diversity of cellular activities, including embryonic stem-cell development, tissue regeneration, cell differentiation, and immune cell regulation [41]. The RAS signaling regulates cancer cell proliferation, apoptosis, inflammation, migration, and metastasis [42]. The aberrant RAS signaling may cause carcinogenesis and targeting it may bring substantial improvement in clinical outcome in metastatic colorectal cancers [43]. It is well established that mTOR signaling, a critical protein in regulating cancer cells, takes part in varieties of fundamental biological activities containing cell death, autophagy, metabolic reprogramming, cell growth, and cell cycle [44]. HIF-1, a transcription factor with important part in response to low oxygen concentrations, or hypoxia, play significant roles in normal tissue development and function and cancer progression [45]. Activation of the HIF-1 pathway has been considered as an attractive therapeutic target [46]. The Hippo signaling, made up of a core kinases module and transcriptional activation module, has recently emerged as an important biochemical signaling in regulating a variety of signaling processes such as organ development and maintenance of tissue homeostasis [47]. The mechanism of miR-200s affecting the metastasis and unfavorable prognosis of CRC patients may be partly understood.

PPI network analysis was applied to investigate the most important target genes for miR-200s in CRC. The top network nodes with high degrees of betweenness centrality and closeness centrality and degree centrality were considered as the hub genes. To further validate the potential roles of the hub genes in CRC, KEGG pathway enrichment analysis was conducted. Results from KEGG analysis demonstrated that these hub genes were also significantly associated with some vital pathways. Most of the pathways have been proved by previous biological experiments. Aside from the above mentioned pathways, Notch signaling pathway has been identified as a highly conserved system in various multicellular organisms that is crucial for driving T-cell fate decisions, proliferation, and aberrant growth [48]. Moreover, VEGF signaling is a pivotal coordinator in the development of tumor angiogenesis, and inhibiting VEGF may provide a promising approach for tumor vessel normalization [49]. From the findings of the PPI analysis, it might be possible to hypothesize that miR-200s have a role in the initiation and progression of CRC by targeting these hub genes involved these key pathways, which may provide potential therapeutic targets.

The network module may determine the stability and function of the network. Correspondingly, the most significant modules of the networks for the targets of miR-200s were identified. KEGG pathway analysis revealed that the genes consisting of the networks for miR-200a, miR-200b and miR-200c were mainly associated with a series of well-studied pathways. In addition to the majority of these pathways that proved by literature exploration, another part have to mention is that the well-studied cell cycle pathway has been a vital pathway mediating every aspect of various fundamental cellular processes such as cell proliferation, apoptosis, differentiation, and migration [50]. Module analysis demonstrated that miR-200s were highly associated with the initiation and development of CRC again.

CRC is a heterogeneous disease and its initiation and progression may be affected by various confounding factors. It is deserved to be mentioned that miR-200a, miR-200b and miR-200c were enriched into the similar functional ontology, pathways, modules or networks and afterwards become more consistent, although they located at different chromosomal regions and regulated different types of genes. By convention, function associated miRNAs may exhibit a coordinated expression to play their role within the same functional modules, suggesting that miR-200a, miR-200b and miR-200c could have a synergistic effect to serve as an effective biomarker during the occurrence and development of CRC [51].

It should be noted that there were several limitations in our study. First, although we have conducted a comprehensive and systematic literature search and enrolled as many documents as possible, the size of available samples for evidence synthesis was still relatively small. More relevant studies and patients would be necessary to improve the reliability and confidence of our findings. Second, the cutoff definitions for high miR-200s expression were not precisely acknowledged among the studies, which could cause potential heterogeneity. Third, although we had planned to consider the impact of several important factors concerning heterogeneity such as age, sex and stage of cancer, this could not be accomplished due to the lack of access to the original data from the reviewed studies. Fourth, as drug resistance is a major problem with most of the cancer treatments. However, the enrolled studies in our study have not focused on the status of miR-200s in those CRC patients who developed resistant to therapy. Moreover, more and more findings have demonstrated that right-sided colon cancers and left-sided colon cancers are distinct clinical and biological entities and suggest that they should be treated as different diseases. However, most of the enrolled studies have not separated the status of miR-200s in tissue samples from different regions of colon in CRC patients. Lastly, our study certainly has its weaknesses due to the lack of experimental validation as such an experiment that focuses on the confirmation of the predictive power of our approach is difficult for us by now.

Nevertheless, we also need to stress some advantages in the present study. We combined two mainstream approaches including meta-analysis and bioinformatics analysis to answer whether miR-200s were associated with the prognosis of CRC patients and why miR-200s could be used as a prognostic biomarker for CRC. Several interesting results from our study may promote further investigation into the functions and partners of miR-200s, push forward the clinical application of miR-200s and also provide us with more targets and strategies for therapy. Although not perfect, most of the findings from our results have been successfully confirmed by recent experimental literatures [34–51]. To better confirm the predictive power of our approach and understand the mechanisms underlying the initiation and progression of CRC, more large-scale and standard investigations are worth conducting.

## Conclusions

In conclusion, based on meta-analysis and bioinformatics analysis results, our study demonstrated that miR-200s could be promising biomarkers for CRC. However, further molecular and functional studies and large-scale controlled clinical studies are required to further explore the biomarker roles of miR-200s.

## Supplementary information

**Additional file 1: Table S1.** miRNAs associated with CRC patient survival.

## Data Availability

The data supporting the conclusions of this article is within the article.

## Abbreviations

CRC: Colorectal cancer
CT: Computed tomography
TNM: Tumor node metastasis
HR: Hazard ratio
CI: Confidence interval
PRISMA: Preferred Reporting Items for Systematic Reviews and Meta-Analyses
NOS: Newcastle-Ottawa Scale
GO: Gene ontology
KEGG: Kyoto Encyclopedia of Genes and Genomes
PPI: Protein–protein interaction
DAVID: Database for annotation, visualization, and integrated discovery
