# Supplementary material for: Integrated characterization and validation of the prognostic significance of microRNA-200s in colorectal cancer
Source: Cancer Cell Int. 2020 Feb 18;20:56. doi: 10.1186/s12935-020-1142-1 (PMC7029504; doi:10.1186/s12935-020-1142-1)
Supplement: Supplementary file 1 — Additional file 1: Table S1. miRNAs associated with CRC patient survival. [file 12935_2020_1142_MOESM1_ESM.docx]

Table S1 miRNAs associated with CRC patient survival

| miRNA | Reference (PMID) | miRNA | Reference (PMID) |
| --- | --- | --- | --- |
| let-7a | 27881005 | miR-203 | 27376958 |
| let-7b | 27126129 | miR-204 | 25209181 |
| let-7c | 24503111 | miR-206 | 26406866 |
| let-7e | 28262692 | miR-20a | 27247088 |
| let-7g | 23932154 | miR-21 | 28376502 |
| let-7i | 25663689 | miR-210 | 24632577 |
| miR-100 | 25216869 | miR-211 | 26152286 |
| miR-101 | 23121918 | miR-212 | 23583431 |
| miR-103a | 27602163 | miR-214 | 24616020 |
| miR-106a | 26250939 | miR-215 | 23532818 |
| miR-106a | 26224446 | miR-217 | 26016795 |
| miR-106b | 25755775 | miR-218 | 24294377 |
| miR-107 | 25197016 | miR-22 | 22492279 |
| miR-10b | 25663689 | miR-221 | 25075256 |
| miR-122 | 27632639 | miR-223 | 25270282 |
| miR-124 | 25081869 | miR-224 | 23846336 |
| miR-124 | 25197016 | miR-229 | 25197016 |
| miR-125b | 24746948 | miR-23b | 28101227 |
| miR-126 | 22397399 | miR-24 | 25502080 |
| miR-1260 | 27399918 | miR-25 | 24293092 |
| miR-128 | 24046120 | miR-26a | 27974852 |
| miR-1288 | 24009195 | miR-26b | 27401248 |
| miR-1290 | 27502702 | miR-296 | 28209128 |
| miR-1292 | 28054337 | miR-29a | 22018950 |
| miR-1297 | 27279909 | miR-29b | 26318304 |
| miR-130a | 27974852 | miR-30a | 25582198 |
| miR-130b | 24027433 | miR-30b | 24293274 |
| miR-132 | 24914372 | miR-30d | 28440426 |
| miR-133a | 25104873 | miR-31 | 24771647 |
| miR-133b | 21573504 | miR-32 | 24123284 |
| miR-134 | 26897940 | miR-320 | 18676867 |
| miR-135 | 27878288 | miR-324 | 24940606 |
| miR-135b | 24735923 | miR-326 | 25760058 |
| miR-137 | 23275153 | miR-328 | 27881005 |
| miR-138 | 24171926 | miR-335 | 24829139 |
| miR-139 | 25550849 | miR-337 | 25197016 |
| miR-140 | 25980495 | miR-338 | 24824250 |
| miR-141 | 24510588 | miR-33b | 26329295 |
| miR-143 | 27247088 | miR-340 | 24448820 |
| miR-144 | 22983984 | miR-342 | 25075256 |
| miR-145 | 27071407 | miR-345 | 24940606 |
| miR-148a | 26389729 | miR-34a | 25362853 |
| miR-149 | 22821729 | miR-361 | 25965817 |
| miR-150 | 22052060 | miR-362 | 23280316 |
| miR-153 | 23950211 | miR-365 | 22072615 |
| miR-154 | 26048406 | miR-370 | 25197016 |
| miR-155 | 21412018 | miR-372 | 22456107 |
| miR-15a | 28405803 | miR-376a | 25422250 |
| miR-15b | 26743779 | miR-378 | 24412052 |
| miR-16 | 24045965 | miR-422 | 27350737 |
| miR-17 | 22065543 | miR-424 | 28054337 |
| miR-17-3p | 26250939 | miR-429 | 24237355 |
| miR-181a | 23023298 | miR-450 | 27494869 |
| miR-181b | 23719259 | miR-4500 | 27686621 |
| miR-181c | 28036302 | miR-455 | 25329796 |
| miR-182 | 23474644 | miR-4772 | 27788488 |
| miR-1826 | 25968874 | miR-4775 | 28095858 |
| miR-183 | 24150523 | miR-487 | 28000854 |
| miR-185 | 21573504 | miR-490 | 26714817 |
| miR-187 | 27329595 | miR-491 | 25075256 |
| miR-188 | 27601590 | miR-494 | 25270723 |
| miR-18a | 23437304 | miR-498 | 18676867 |
| miR-191 | 24195505 | miR-503 | 28054337 |
| miR-193 | 25232258 | miR-505 | 25197016 |
| miR-194 | 26318304 | miR-506 | 26452129 |
| miR-194 | 25285168 | miR-515 | 27092493 |
| miR-195 | 28356122 | miR-517 | 27073521 |
| miR-196a | 25525411 | miR-556 | 25788261 |
| miR-196b | 25329796 | miR-570 | 26389729 |
| miR-197 | 27881005 | miR-573 | 27092493 |
| miR-198 | 25174450 | miR-579 | 27092493 |
| miR-199a | 23292866 | miR-590 | 27735951 |
| miR-199b | 27145368 | miR-592 | 25661360 |
| miR-19a | 26057451 | miR-625 | 23861214 |
| miR-19b | 21722265 | miR-628 | 24940606 |
| miR-200a | 24504363 | miR-630 | 24981248 |
| miR-200b | 27632639 | miR-638 | 25301729 |
| miR-200c | 23982750 | miR-652 | 27881005 |
| miR-200c | 18079988 | miR-664 | 25329796 |
| miR-203 | 26701878 | miR-6826 | 27878288 |
| miR-6875 | 27878288 | miR-9 | 25940709 |
| miR-7 | 27126129 | miR-92a | 23625654 |
| miR-720 | 25286763 | miR-92a | 22772712 |
| miR-802 | 27092493 | miR-93 | 23354160 |
| miR-875 | 27302926 | miR-944 | 23280316 |
| miR-885 | 25663689 | miR-96 | 27044381 |
| miR-886 | 24940606 | miR-99a | 28130225 |
| miR-889 | 25197016 | miR-99b | 26259252 |
